# Single-cell splicing QTL analysis in pancreatic islets

**DOI:** 10.3389/fbinf.2025.1657895

**Published:** 2025-09-10

**Authors:** Jae-Won Cho, Jingyi Cao, Martin Hemberg

**Affiliations:** The Gene Lay Institute of Immunology and Inflammation, Brigham and Women’s Hospital, Massachusetts General Hospital, and Harvard Medical School, Boston, MA, United States

**Keywords:** alternative splicing, single-cell RNA-sequencing, pancreatic islet, splicing quantitative trait loci, CDC42

## Abstract

**Introduction:**

Alternative splicing (AS) of mRNAs is a highly conserved mechanism which can greatly expand the functional diversity of the transcriptome. Aberrant splicing underpins many diseases, and a better understanding of AS can provide insights regarding the molecular mechanisms involved. Importantly, AS can be affected by genetic variants and several studies have indicated large numbers of splicing quantitative trait loci (sQTL). With the advance of single-cell technology, expression QTL studies have been expanded to identify cell type level variants.

**Methods:**

We collected eight full-length scRNA-seq pancreatic islet datasets. Genotyping for each individual was done by the CTAT pipeline and Streka2. The isoform quantification was done by RSEM. Finally, sQTL was obtained by sQTLseeker2.

**Results:**

As a result, we identified 228 cell type level sQTLs for alpha and beta cells across 152 genes. In particular, our study highlights four variants affecting CDC42, a gene related to cell morphology, which have not been observed from bulk sQTL analysis.

**Discussion:**

Our results provide a proof of concept that it is possible to identify cell type level sQTLs, and we envision that better powered studies will allow us to further uncover the genetic regulation of splicing.

## 1 Introduction

Alternative splicing (AS) is pervasive in the human genome, and it allows for a remarkable expansion of protein diversity (Black, 2000; Barrie et al., 2012). AS is a highly conserved mechanism, and aberrant splicing has been implicated in a wide range of diseases (Lopez-Bigas et al., 2005; Feng and Xie, 2013; Wang and Lee, 2018; Escobar-Hoyos et al., 2019). A better understanding of AS will not only allow us to appreciate the role of transcriptome diversity, but it could also inform the development of novel therapies.

One successful strategy for identifying functional mechanisms and potential drug targets has been through naturally occurring genetic variants. Specific variants can be linked to a trait through genome-wide association studies (GWAS) (Manolio, 2010; Sollis et al., 2023). In addition, variants have also been linked to molecular phenotypes and expression quantitative trait loci (eQTL) analysis has been widely used to show a link between SNPs and gene expression (Carithers and Moore, 2015; Consortium et al., 2017). Similarly, splicing QTL (sQTL) analysis has been used to link SNPs to the alteration of splicing events (Monlong et al., 2014; Garrido-Martin et al., 2021; Yamaguchi et al., 2022). Previously, more than 30 studies with 127 datasets were reported in the 2023 eQTL catalogue (Kerimov et al., 2023) which contains information from >7,500 donors and >1.7 million fine mapped associations. There are also several splicing studies, including GTEx (Zhang et al., 2020; Garrido-Martin et al., 2021; Atla et al., 2022; Brotman et al., 2022; Qi et al., 2022; Yamaguchi et al., 2022) which contains >210,000 sQTLs from >600 donors.

With the advent of single-cell technologies, eQTL studies have been expanded to reveal cell type specific variants (van der Wijst et al., 2020; Cuomo et al., 2021; Neavin et al., 2021; Nathan et al., 2022; Yazar et al., 2022). Neavin et al. (2021) studied single-cell eQTLs in fibroblasts and fibroblast derived iPSCs from a total of 110 donors, and they found 71,926 reprogramming related eQTLs. They observed numerous cell type-specific eQTLs, while only 41.1% were observed in bulk-eQTL results from GTEx. Yazar et al. (2022) conducted single-cell eQTL analysis with PBMCs collected from 982 donors, and found loci related to the autoimmune disease at the cellular level. They also observed immune cell-specific eQTLs, of which only 40%–63% were observed in bulk PBMC data. Nathan et al. (2022) studied single-cell eQTLs in memory T cells with 259 individuals, and they also reported cell state specific eQTLs, of which 80%–90% were observed from bulk data.

However, cell-type level sQTLs have not yet received the same amount of attention. The main reason why this has not been attempted previously is because sQTL studies require both a sufficient number of samples as well as good coverage of splice junctions. A sufficient sample size is critical for those QTL analyses since previous analyses using bulk data in the GTEX database (Shan et al., 2019) have shown that the number of significant eQTLs is strongly correlated with the sample size (Supplementary Figure S1A), indicating that the number of donors is a key factor for sQTL analysis. Although there are studies profiling a large number of individuals, they employ single cell technologies that detect only the 3′- or 5′-ends of each transcript, and thus there is not enough information to quantify splice junction usage (Arzalluz-Luque and Conesa, 2018; Westoby et al., 2018). Despite this limitation, a recent study reported splicing QTL at the single-cell level using 5′-end 10X scRNA-seq for PBMC data. The authors focused on ancestry-related sQTL,the reads that were biased to the 5′ region, which may lead to poor coverage for detecting other splicing events, something the authors mentioned as a limitation (Tian et al., 2024). Since the platforms that are capable of profiling full-length transcripts are more expensive, there are not many datasets suitable for sQTL analyses available. An additional obstacle for sQTL analyses comes from the fact that donor genotypes are required. Even though the gold standard is to use WGS or a chip to profile each donor, genotyping by using full-length scRNA-seq was recently reported, and benchmarks demonstrated an overall F1-score >0.95 (Liu et al., 2019). Moreover, it has been shown that cell type specific splicing events can be reliably identified via full-length scRNA-seq (Westoby et al., 2018). Combining these approaches, we collected eight pancreatic islet datasets to obtain a sample size sufficient for performing single-cell sQTL analysis. From a previous bulk RNAseq study of sQTLs in pancreatic islets which used 399 human donors, 4,858 sQTLs from 2,058 sGenes were reported (Atla et al., 2022). Based on the association between those sQTLs and known T1D or T2D risk variants, the authors concluded that there is a functional association of T1D or T2D disease due to aberrant splicing events. The authors also observed an association between sGenes and fatty acid biosynthesis (Armour et al., 2023) or mTORC1 pathway (Blandino-Rosano et al., 2012; Blandino-Rosano et al., 2017; Asahara et al., 2022). These implications are important since these pathways had previously been associated with cell types in pancreatic islets. By using single-cell level sQTL analysis, we can further link those sQTLs in a cell type-specific manner.

## 2 Methods

### 2.1 Data acquisition

The dataset for islet atlas was obtained from Camunas-Soler et. al. (islet1) (Camunas-Soler et al., 2020), Segerstolpe et. al. (islet2) (Segerstolpe et al., 2016), Enge et. al. (islet3) (Enge et al., 2017), Xin et. al. (islet4) (Xin et al., 2016), Dorajoo et. al. (islet5) (Dorajoo et al., 2017), Lawlor et. al. (islet6) (Lawlor et al., 2017), Wang et. al. (islet7) (Wang et al., 2016), and Marquez-Curtis et. al. (islet8) (Marquez-Curtis et al., 2022). For islet1, we excluded 3 T1D patients due to the small sample size. We excluded the R269 sample since the raw data was not available. For islet3, we only used adult samples. For islet5, we excluded “subject 3” sample since the raw data was not available. For islet7, we excluded HP15041 due to genotyping problems. We used cell-type information from the original papers. For islet6, we used marker genes threshold for cell typing based on Supplementary Table S3 in the original paper. We only considered major islet cell types: alpha, beta, gamma, delta, acinar, and ductal cells.

### 2.2 Genotyping

We used the Trinity Cancer Transcriptome Analysis Toolkit (CTAT) which is based on the GATK Best Practices pipeline (Liu et al., 2019; Brouard and Bissonnette, 2022) for the variant calling of single cells in scRNA-seq data (Fangal, 2020). We ran the pipeline with either “single-end” or “paired-end” for each corresponding dataset. We set the parameter “boosting_method”, which speeds up during the variant calling, to be “none”. We excluded SNVs from the known RNA-editing sites from RADAR (Ramaswami and Li, 2014) and RediProtal (Picardi et al., 2017) databases, which were provided by the CTAT pipeline. During the process, 13/1,464 islet1 cells, 24/2,038 islet2 cells, 13/1,578 islet3 cells, 13/1,507 islet4 cells, 222/333 islet5 cells, 9/593 islet6 cells, 59/268 islet7 cells, and 1/147 islet8 cells were excluded due to lack of confident SNVs or absence of raw data from a certain sample. After obtaining all the SNVs from single cells, we merged per donor using bcftools (1.13) (Danecek et al., 2021). Since somatic mutations could exist, we ran a germline mutation calling pipeline from Strelka2 (2.9.2) (Kim et al., 2018) with merged bam files (using Samtools; v1.11) (Danecek et al., 2021) for each donor to identify germline mutations. We only keep the “passed” germline mutation from Strelka2 overlapping the merged mutation profiles at the donor level from the CTAT pipeline. We only keep the genotype information if there is a read at that region. We excluded X, Y, and M chromosomes. We excluded variants with minor allele frequency (MAF) < 0.1 (genomicMateSelectR v0.2.0) (Wolfe, 2022) from both healthy individuals and T2D patients. Locational information for SNP was obtained from CTAT result.

### 2.3 Transcript and gene quantification

We obtained the transcript and gene expression matrix by running RSEM (Li and Dewey, 2011; Westoby et al., 2018) (1.3.0) using rsem-calculate-expression--estimate-rspd--no-bam-output -p 16 with GRCh37 version 19 (Ensembl 74) (transcript file: *.isoforms.results, gene file: *.genes.results). As a result, we could align reads to 38,381 transcripts and 20,392 genes. The mean number of expressed genes per cell was 6,196 and the mean number of expressed transcripts per cell was 6,835.

### 2.4 Genomic location of transcript

We extracted genomic region of each gene by GenomicFeatures package (v 3.17) with [type = = “gene”, gene_status ! = “PUTATIVE”, no duplicated gene name] (Lawrence et al., 2013).

### 2.5 scRNA-seq processing

We used log2 (TPM + 1) matrix of transcript from RSEM for running Seurat (v 4.1.0) (Butler et al., 2018)pipeline (FindVariableFeatures, ScaleData, RunPCA, RunUMAP [dims = 1:30]). We corrected batch effect of different dataset by running Harmony (v 0.1.0) (Korsunsky et al., 2019). We performed clustering by FindNeighbor (reduction = “harmony”, dims = 1:30) and FindClusters (resolution = 1.2). We excluded misaligned cell type from each cluster to remove batch outliers. We generated pseudobulk cell type-specific transcript matrix by averaging the transcript expression level per sample. After that, we used inverse log transformation (2^x −1) for sQTLseeker2 input since they suggested using a non-log transformed matrix to better calculate Hellinger distance for transcript expression.

### 2.6 sQTL analysis

We used the sQTLseekeR2 (v1.1.0) (Garrido-Martin et al., 2021) package for cell type level sQTL. We filtered out the lowly expressed transcript and gene by “min.transcript.exp = 0.1” and “min.gene.exp = 0.1” using prepare.trans.exp function. We used sqtl.seeker function to obtain nominal p-value with “svQTL = TRUE” (to remove a potential false positive sQTL by inhomogeneity in the variance from different genotype groups), “genic.window = 50,000 (bp)”, “min.nb.ind.geno = 5 (at least five samples for a certain genotype)”, “ld.filter = 0.8 (Linkage disequilibrium threshold)”. Age and gender were used for covariables to regress the transcript expression level. For multiple hypotheses correction, we used sqtls function with “FDR = 0.1” and “FDR.svQTL = 0.01” with default parameters (md.min: the minimum MD (Maximum Difference) in relative expression: 0.05).

### 2.7 Differential expressed gene analysis

For DEG analysis, we normalized the gene expression from the Seurat package (v 4.1.0) (Butler et al., 2018) and performed the FindMarkers function with logfc.threshold = 0.25 and min.pct = 0.1. We further used p.adjusted < 0.05.

### 2.8 eQTL analysis

We used FastQTL (v2.184) (Ongen et al., 2016) to obtain cell type level eQTL with only the SNPs tested for sQTL analysis. The same covariables were used with “--window 1e7” parameter setting. The gene expression matrix was obtained by the same process as the transcript matrix. We only kept eQTLs with q-value < 0.1.

### 2.9 RBP binding site

We obtained RNA binding protein (RBP) site from POSTAR3 (http://111.198.139.65/index.html) (Zhao et al., 2022) with only the “experimental results”. We obtained RBP overlapping SNP if the SNP is located within the RBP binding region. We only considered an RBP if it is expressed in the cell type (log2 (exp+1) > 0.25 and fraction of cells expressing > 0.1).

### 2.10 GWAS analysis

To analyze the association between GWAS related to pancreas disease and SNPs from sQTL, we collected “type 2 diabetes mellitus” (EFO ID: MONDO_0005148), “type 1 diabetes mellitus” (EFO ID: MONDO_0005147), and “pancreatitis” (EFO ID: EFO_0000278) from GWAS catalog (https://www.ebi.ac.uk/gwas/) (Sollis et al., 2023). We obtained “glycemic traits” from Ji et al. (Chen et al., 2021). Since GWAS SNPs were in hg38 coordinates, we converted hg38 genome coordinate into hg19 by LiftOver (https://liftover.broadinstitute.org/) (Hinrichs et al., 2006). Unknown alleles were subsequently removed. We obtained LD > 0.6 SNPs from each GWAS by SNiPA (setting: GRCh37.1000 genomes, Phase 3 v5, European, Ensembl 87) (https://snipa.org/snipa3/) (Arnold et al., 2015).

### 2.11 Splicing type analysis

We used the “splicing events classification” pipeline from sQTLseekeR (v2.1) (Monlong et al., 2014).

### 2.12 Pathway analysis

We used EnrichR (Xie et al., 2021) with KEGG (Kanehisa, 2019), NCI-Nature (Schaefer et al., 2009), and Gene ontology (Gene Ontology et al., 2023). We required FDR < 0.1 for a pathway to be considered significant.

## 3 Results

### 3.1 Overview of the single-cell sQTL analysis

**FIGURE 1:**
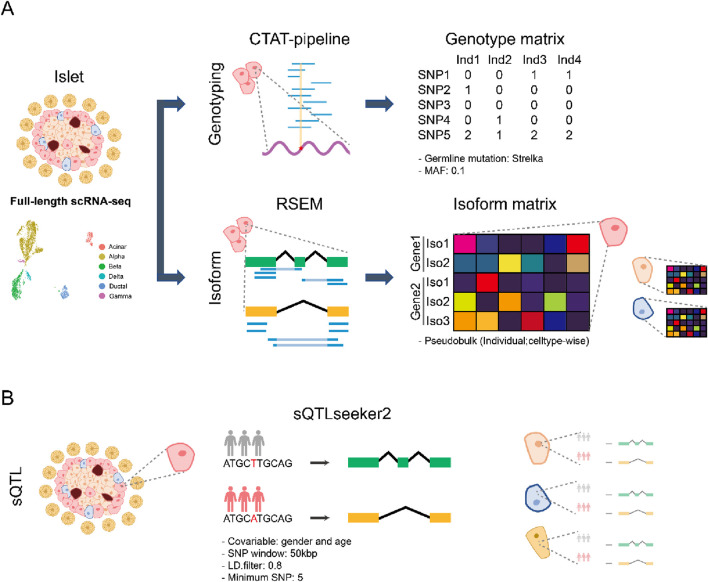
Overview of the single-cell sQTL analysis **(A)** Schematic workflow of preprocessing of islet datasets. Full-length scRNA-seq from eight pancreatic islet datasets were collected. The genotype for each individual was obtained by running the CTAT pipeline for each cell. After merging the SNP information across donors, we only retained germline mutations identified by Strelka. Furthermore, MAF < 0.1 SNPs were excluded. RSEM was used for transcript quantification, and pseudobulks for each individual were subsequently generated. **(B)** Schematic workflow of sQTL identification for pancreatic islet datasets. For each cell type, we ran sQTLseeker2 to obtain celltype-specific sQTL. During the analysis, we used gender and age as covariate.

We collected eight full-length scRNA-seq pancreatic islet datasets (Segerstolpe et al., 2016; Wang et al., 2016; Xin et al., 2016; Dorajoo et al., 2017; Enge et al., 2017; Lawlor et al., 2017; Camunas-Soler et al., 2020; Marquez-Curtis et al., 2022; Supplementary Table S1), comprising of 4,841 cells from 54 healthy individuals and 2,574 cells from 21 type 2 diabetes (T2D) patients divided amongst six cell types (acinar, alpha, beta, gamma, delta, and ductal cells) (Supplementary Table S1). We used the CTAT pipeline (Fangal, 2020), which is based on the STAR aligner and the GATK-best practice variant calling pipeline. Importantly, it has been shown to perform well in a benchmark for genotype inference from scRNA-seq (Liu et al., 2019). To infer the genotype we combined all the mutation profiles from each cell within each individual. Since these profiles may contain somatic mutations, we only keep the germline variants predicted by Strelka2 (Kim et al., 2018). We further filtered out the SNPs with minor allele frequency (MAF) < 0.1. As a result, we could obtain ∼24,000 SNPs per individual, similar to the ∼35,000 SNPs that would be expected from exome sequencing (Marian, 2014) (Supplementary Tables S1, S2) The lower number of SNPs is expected since we are restricted to the genes that are expressed in our sample. For transcript quantification we used RSEM (Li and Dewey, 2011) since it has been reported as the best performing tool for isoform level expression quantification with full-length scRNA-seq data (Westoby et al., 2018). Prior to clustering we ran Harmony (Korsunsky et al., 2019) to correct the batch effect from healthy donors or T2D patients, respectively. Then, we removed 416 cells from the healthy and 283 cells from the T2D group that had been aligned to different cell types from the original paper, assuming they were batch outliers (Supplementary Figure S2; Supplementary Table S3). For cell type level sQTL analysis, we created pseudobulks for each cell type following the same strategy used in single-cell eQTL analysis (Cuomo et al., 2021; Yazar et al., 2022). However, due to the small number of cells, we only analyzed alpha, beta, delta, and ductal cells (Figure 1A). We also created a pseudobulk of pancreatic islets called “collapsed” to mimic bulk pancreatic islet data (Supplementary Table S4).

After preprocessing, we used sQTLseeker2 (Garrido-Martin et al., 2021) to obtain cell type level sQTLs (Figure 1B). We followed the recommendations by the authors of sQTLseeker2 and filtered out the lowly expressed transcripts. We applied a linear model to adjust the expression levels, using age and gender as covariates. A 50 kbp window centered around each splice junction was used to identify candidate variants since previous studies have shown that most sQTLs occur within this distance, while larger windows reduce power due to multiple-hypothesis correction (Garrido-Martin et al., 2021; Atla et al., 2022; Brotman et al., 2022). The number of variants tested was further reduced by merging those that had a linkage disequilibrium > 0.8. Not all cell types were annotated in all datasets, and for each cell type we had at least 15 individuals per genotype.

Our analysis yielded 136 sQTLs from alpha cells, 92 from beta cells, while only 1 or 2 sQTLs were significant in other cell types at an FDR of 0.1. For the collapsed dataset we identified 268 sQTLs (Supplementary Tables S5, S6). Previous analyses using bulk data in the GTEX database (Shan et al., 2019) have shown that the number of significant eQTLs is strongly correlated with the sample size (Pearson correlation coefficient: 0.86) (Supplementary Figure S1A). We also observed a similar pattern from our data by down-sampling (Supplementary Figure S1B). We assumed a similar relationship exists for sQTLs, and extrapolating from a brain study, the expected number of sQTLs from 54 samples is ∼300 (63 samples: 387, 124 samples: 849) (Zhang et al., 2020), similar to the 268 sQTLs that we observed in the collapsed data. Similarly, a study of sQTLs using bulk RNAseq from pancreatic islets (Atla et al., 2022), reported 4,858 sQTLs from 400 samples using an FDR of 0.01. Assuming that the number of sQTLs scales linearly with the sample size, then 268 sQTLs for 54 samples is an expected result. Taken together, these comparisons show that the number of sQTLs from the “collapsed” data was within the expected range. For T2D patients, due to the sample size, we could not get any suitable number of significant results (Supplementary Table S5).

**FIGURE 2:**
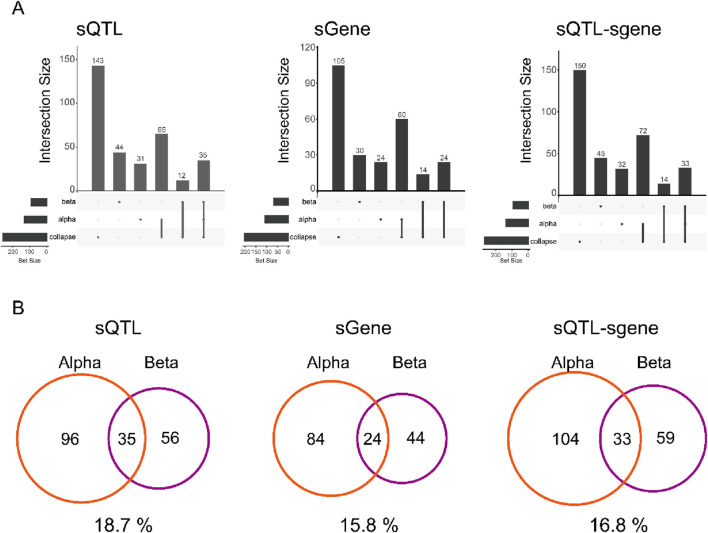
Cell type-specific sQTLs in islet **(A)** UpSetR plot of sQTL, sgene, and sQTL-sgene pair between alpha cell, beta cell, and collapsed sample. Each element represents a SNP, which has been merged by LD block. **(B)** Venn diagram of overlap between alpha and beta cells for sQTL, sgene, and sQTL-sgene pair. Overlap % is shown below each Venn diagram.

We also note that the effect sizes are often substantial. For both alpha and beta cells, > 92% of sQTLs result in a change in a maximum difference of relative abundance > 0.2 which is considered a large effect size in the sQTLseeker2 paper (Garrido-Martin et al., 2021). Comparing the distribution of sQTLs across cell types we found a substantial number of sQTLs and genes showing the alteration in the splicing affected by these variants (sgenes) that do not overlap with the “collapsed” result (Figure 2A). This result is consistent with single cell eQTL studies which have shown that bulk transcriptomes can mask the signal from individual cell types. For both alpha and beta cells, the overlap was only around 15%–20%, indicating that AS in different cell types is regulated by different SNPs (Figure 2B). At the same time, the splicing of a given gene was also affected differently between different cell types with only 15.8% of sgenes found in both alpha and beta cells, suggesting that the set of sQTLs affecting a given pathway will differ between cell types.

Interestingly, the expression levels of sgenes are typically not higher in the cell type where they are active. We performed differentially expressed gene (DEG) analysis between alpha and beta cells to obtain highly expressed genes for each cell type. The 32% for alpha cells and 20% for beta cells is the ratio of the DE genes among sgenes. However, we only found 1.7% of highly expressed genes in the alpha cell (27/1,626), and 0.32% of highly expressed genes in the beta cell (9/2,780) were cell type-specific sGenes. Hence, this implies that DE genes are distinct from sgenes.

### 3.2 Functional analysis of sQTL

**FIGURE 3:**
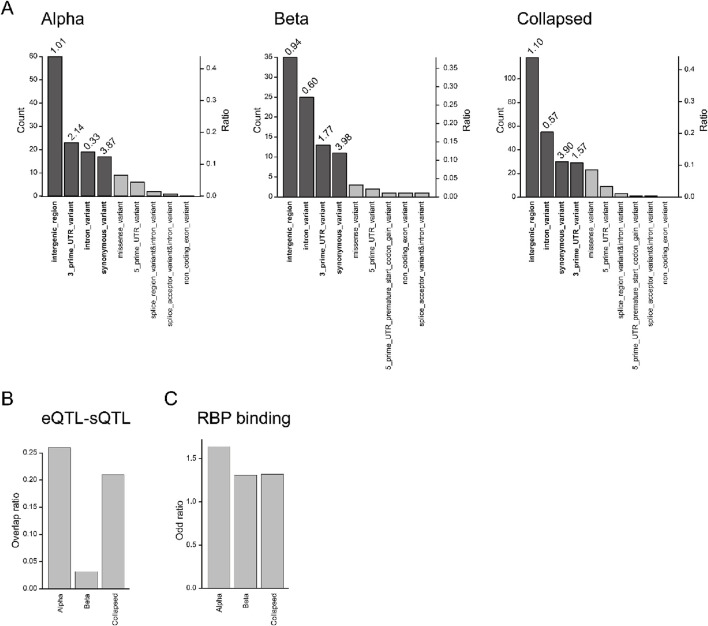
Functional analysis of sQTLs **(A)** Location of sQTLs in alpha cells, beta cells, and collapsed sample. The number of each category is shown on the left axis, while the proportion of each category is shown on the right axis. The darker shades indicate regions above the threshold for analysis (count >10 and ratio > 0.1). The odd ratio of each category above the threshold is shown on the top of each bar. **(B)** The overlap ratio between sQTLs and eQTLs in each cell type. **(C)** The odd ratio of the RBP binding site with the sQTLs in each cell type.

Next, we characterized the sQTLs’ genomic locations (Figure 3A; Supplementary Table S7; Fangal, 2020), and this revealed that most sQTLs were located outside of exonic regions, with only 26/137 alpha cell variants and 15/92 beta cell variants overlapping exons. Comparing each region to the total number of detected variants suggested that SNPs from intergenic regions were not enriched above chance level since the odd ratios were close to 1. A previous study of sQTLs in the pancreatic islet showed that 5′ and 3′ UTRs were the most enriched regions (Atla et al., 2022). Although we could not find as many sQTLs from the 5′ UTR as in the previous report, we observed substantial numbers of sQTLs in 3′ UTR (odd ratios, alpha: 2.14, beta:1.77, collapsed: 1.57). We also observed a higher number of sQTLs in intronic regions which was not surprising given its key role in splicing (Jo and Choi, 2015), but the odd ratios were surprisingly low (alpha: 0.33, beta: 0.7, collapsed: 0.57). Although the splice site has been shown to be enriched for sQTLs (Garrido-Martin et al., 2021; Atla et al., 2022; Qi et al., 2022), we did not observe this in our study. One explanation could be that the splice junction can only be observed from unspliced transcripts. We also cannot rule out that the discrepancy from previous studies is due to variant calling from the transcripts. Second, we investigated whether sQTLs are associated with eQTLs (Supplementary Table S8). A previous bulk pancreatic islet study only reported a weak association between sQTLs and eQTLs (∼25%) (Atla et al., 2022), suggesting that regulation of gene expression levels and splicing are largely independent. Similarly, when we compared cell type level sQTLs and eQTLs, we found a low overlap (<27%) (Figure 3B), suggesting that gene expression and splicing regulation are largely independent also at the cell type level. Third, we hypothesize that sQTLs would be enriched at RNA binding protein (RBP) (Zhang et al., 2020; Garrido-Martin et al., 2021; Qi et al., 2022) sites. We analyzed whether sQTLs are enriched at RBP sites using the POSTAR3 database (Zhao et al., 2022), and as expected odd ratios were higher than 1 for alpha, beta, and “collapsed” (Figure 3C; Supplementary Table S9). Fourth, since many sQTLs have been associated with disease (Zhang et al., 2020; Garrido-Martin et al., 2021; Atla et al., 2022; Brotman et al., 2022; Qi et al., 2022), we investigated overlap with GWAS variants related to type 1 diabetes (T1D), type 2 diabetes (T2D), and glycemic traits (Atla et al., 2022) within LD > 0.6 SNPs using SNiPA (Arnold et al., 2015). However, we could not observe any GWAS association for our sQTL results, and we conjecture that this is due to the small number of variants identified.

### 3.3 Cell type-specific splicing in sQTL

**FIGURE 4:**
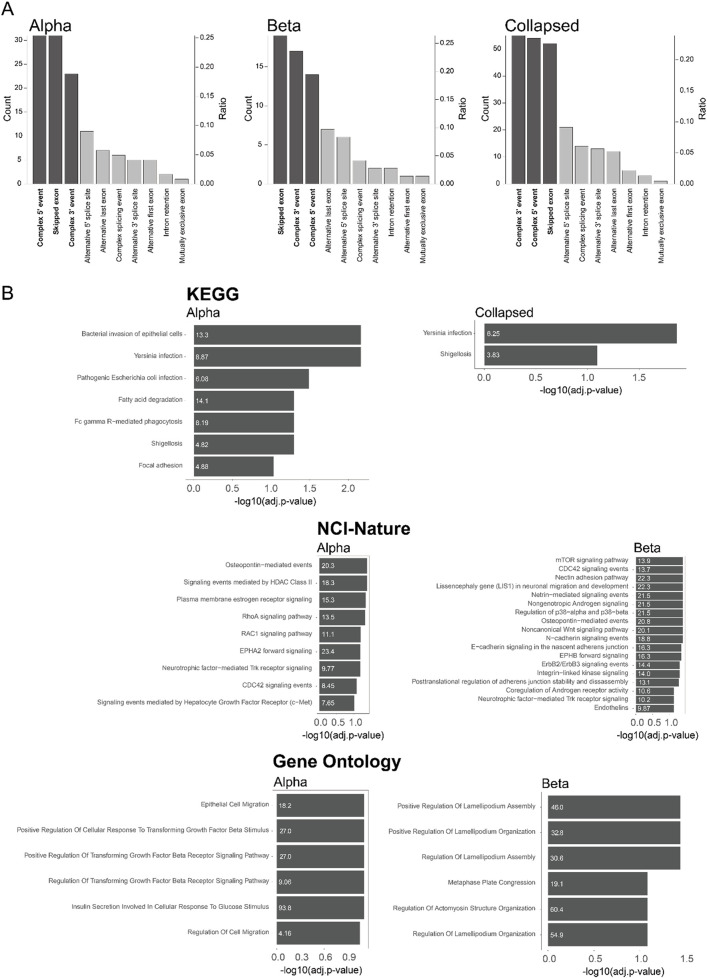
Cell type-specific splicing in sQTL **(A)** Type of event associated with sQTLs in alpha cells, beta cells, or collapsed sample. Unknown splicing type events were excluded. The number of each category is shown on the left axis, while the proportion of each category is shown on the right axis. The dark color indicates the category above the threshold for analysis (count > 10 and ratio > 0.1) **(B)** Pathway enrichment by KEGG, NCI-Nature, and Gene ontology using EnrichR. Only the pathways with adjusted p-value < 0.1 are shown. The odds ratio of pathway enrichment is shown inside the barplots.

Next, we characterized the splicing events involved in sQTL using the definitions from sQTLseeker (Monlong et al., 2014). Most of the splicing events were complex 5′ events, complex 3′ events, and skipped exon events, except unknown events (Monlong et al., 2014; Figure 4A; Supplementary Table S10). This finding was consistent for alpha, beta, and “collapsed”. Compared to previous reports, we observed a similar number of complex 5′ and 3′ events, but we observed more skipped exons (Monlong et al., 2014). We also carried out functional analysis for sgenes using EnrichR (Xie et al., 2021) to quantify the overlap with KEGG (Kanehisa, 2019), NIC-Nature (2016) (Schaefer et al., 2009), and Gene ontology (2023) (Gene Ontology et al., 2023; Figure 4B; Supplementary Table S11). A previous bulk pancreatic islet sQTL study (Atla et al., 2022) showed enrichment for terms related to virus infection (herpes simplex virus 1 infection and viral myocarditis) from the KEGG pathway. Our “collapsed” result was also enriched in bacterial response (*yersinia* infection and shigellosis), indicating an association with the inflammation pathway. Previously, it has been reported that the association between inflammation and pancreatic islets such as microbial DNA mediated pancreatic islet inflammation in obesity (Gao et al., 2022) or bacterial impact on T1D (Abdellatif et al., 2019). Hence, this result indicates that one of the mechanisms through which the genotype impacts obesity or T1D is via changed splicing pattern in response to inflammation. However, only a few pathways were enriched in the “collapsed”, indicating a dilution effect by averaging the signals from different cell types.

On the other hand, there were several enriched pathways from both alpha and beta cells that were found in only one cell type (Figure 4B; Supplementary Figure S3). One interesting finding was that the alpha cells are enriched for inflammation and fatty acid pathways, while the beta cells were enriched for the mTORC1 pathway. Both the association between the alpha cell and fatty acid pathway (Armour et al., 2023), and the association between the beta cell and mTOR signaling (Blandino-Rosano et al., 2012; Blandino-Rosano et al., 2017; Asahara et al., 2022) were reported previously from a bulk study (Atla et al., 2022), indicating cell type specific regulation. The alpha cells were also enriched in TGF-beta signaling, which was surprising since a tight association between beta cells and TGF-beta signaling has been reported previously (Lee et al., 2021; Wang et al., 2022). This result implies an additional functional association of TGF-beta signaling in alpha cells.

**FIGURE 5:**
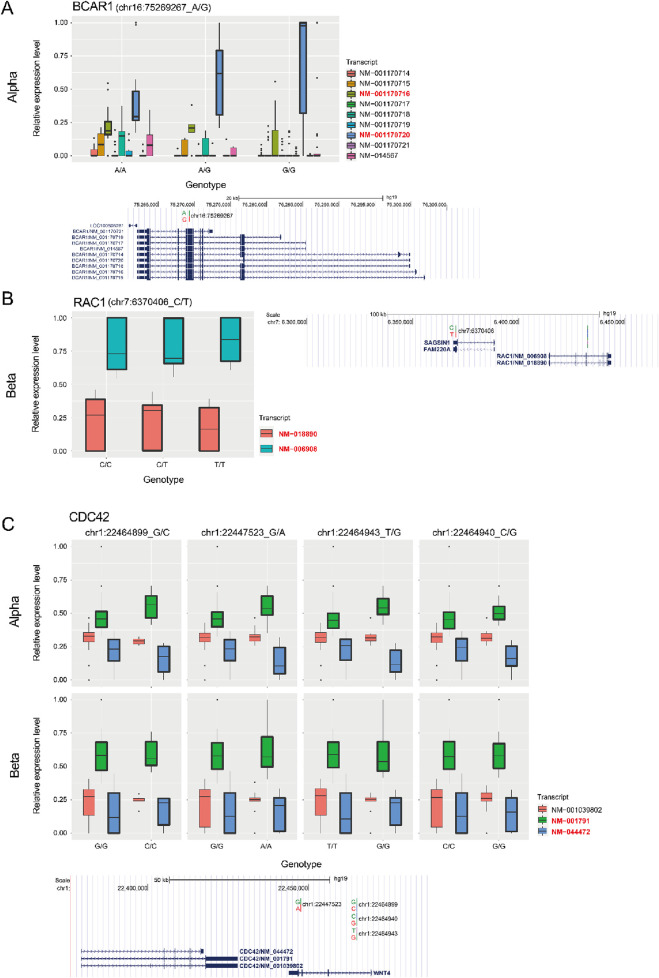
Relative abundance of transcripts in BCAR1, RAC1, and CDC42 genes. **(A)** (*top*) Relative abundance of each transcript (log2 (exp+1)) at the BCAR1 locus between different genotypes in alpha cells. (*bottom*) Genomic location of BCAR1 isoforms with sQTL in the UCSC browser (Clawson et al., 2023) (hg19, http://genome.ucsc.edu). **(B)** (*left*) Relative abundance of each transcript (log2 (exp+1)) at the RAC1 locus between different genotypes in beta cells. (*right*) Genomic location of RAC1 isoform with sQTL. **(C)** (*top*) Relative abundance of each transcript (log2 (exp+1)) at the CDC42 locus between different genotypes in alpha and beta cells. The transcript IDs of the two transcripts that change the most (and symmetrically) are colored red in the legend **(A–C)**. (*bottom*) Genomic location of CDC42 isoforms with four sQTLs.

Another interesting pathway was the cell morphology pathway mediated by CDC42 which was enriched in both alpha and beta cells. It has been reported that CDC42 is related to cytoskeleton and cellular structure (Filipenko et al., 2005; Watson et al., 2017; Pichaud et al., 2019). This suggests that specific variants can alter pancreatic islet morphology via splicing alterations in a cell type specific manner. In addition, it has been reported that the morphology of the pancreatic islet is associated with diabetes (Bonner-Weir et al., 2015; Okajima et al., 2022). Especially, CDC42 was reported as a regulator for not only diabetes, but also insulin secretion and development (Martinelli et al., 2018; Veluthakal et al., 2018; Huang et al., 2019; He et al., 2020). This implies a further association between splicing alteration via different genotypes and pancreatic islet diseases like diabetes. One example of cell type specific splicing regulation of a pathway is given by BCAR1 which was associated with CDC42 only in alpha cells (Figures 4B, 5A; Supplementary Figure S4A). Reassuringly, it was previously reported to affect cell differentiation via CDC42 (Bauer et al., 2021). By contrast, RAC1 was associated with CDC42 only in beta cells (Figures 4B, 5B; Supplementary Figure S4B), consistent with previous reports linking it to pancreatic islet morphogenesis via beta cells (Greiner et al., 2009). However, none of these three genes were observed in previous bulk pancreatic islet sQTLs (Atla et al., 2022). We investigated CDC42 more carefully and found four SNPs associated with this sQTL from alpha and beta cells. Interestingly, even though they influence the same gene, the perturbation in each cell type was different resulting in varying ratios between isoforms depending on the combination of genetic variants (Figure 5C). This result indicates an additional layer of heterogeneity for sQTLs, which will lead to more diversification in the transcriptome in a cell type specific manner.

## 4 Discussion

We carried out a single-cell sQTL analysis of the pancreatic islet, identifying cell type level variants regulating AS. As expected, the single-cell resolution allowed us to identify variants that were not revealed when analyzing bulk samples. Interestingly, we also captured cell type level regulation of splicing event between different genotypes. Reassuringly, we found a coherent regulation of splicing events by sgenes in each cell type which were often related to diabetes, or a phenotype relevant to pancreatic islets (Monlong et al., 2014; Garrido-Martin et al., 2021; Atla et al., 2022; Brotman et al., 2022; Yamaguchi et al., 2022). This indicates that function or phenotype of the pancreatic islet can affect individuals by different splicing events across cell types. In particular, we found a cell type specific sQTL for CDC42, a gene which had previously been related to pancreatic islet morphology, which has not been reported elsewhere.

However, our study has a several limitations. The cohort only included 54 healthy donors, and not all cell types were represented in all donors. The poor representation of some cell types resulted in few significant sQTLs and poor overlap with pancreatic islet related GWAS. Furthermore, we used a relatively loose threshold compared to other more well powered studies. Also, usually nominal p value and empirical p value by permutation test were used together for eQTL or sQTL analysis. Due to lack of the sample size we could not obtain a suitable number of sQTLs by empirical p value. Another limitation is our restricted genotyping, and we can only call variants overlapping expressed transcripts. Due to the low number of immature mRNAs, the number of variants from intronic regions or splicing sites is limited. Moreover, we did not utilize the population structure to regress out such effects or gene expression distribution (Price et al., 2006; Garrido-Martin et al., 2021). However, these regression models have been reported to result in false positives (Dahl et al., 2019), so given our small sample size this may not be such a severe drawback. Although it is possible to impute SNPs (Marchini and Howie, 2010), we did not use this approach since we cannot guarantee the reliability when only using SNPs from the transcriptome.

Nevertheless, comparison to bulk sQTL and single cell eQTL analyses suggest that we are able to detect the expected number of variants. Hence, by increasing the sample size it will most likely be possible to identify more splice affecting variants. Even though the scRNAseq protocols used in this study are designed to capture mature mRNAs, they will frequently profile non-exonic regions derived from nascent transcripts (Hangauer et al., 2013; Agostini et al., 2021). Here, this was an advantage, as it allowed us to discover additional sQTL variants located in these regions. By using WGS for the genotyping, one would most likely obtain even more cell type level sQTLs. Recently, long read technologies have been combined with single cell RNAseq technologies, and this is likely to be a better and more cost effective approach for obtaining information about isoform usage (Rhoads and Au, 2015; Uapinyoying et al., 2020; Huang et al., 2021). In particular, since we showed that variant calling via short full-length is feasible, this provides further evidence that long read sequencing will be useful for sQTL studies since these technologies generally perform better for variant calling (Shiau et al., 2023; Kim et al., 2024; Penter et al., 2024).

The main limiting factor of our study is not the number of cells, but the number of donors. When we randomly downsampled the cells for each donor, there was almost no difference in the number of sQTLs, indicating a marginal effect by cell count per donor (Supplementary Figure S5). Another benefit from a larger and more diverse set of donors is that it will be possible to reduce errors through population-structure level batch correction and batch correction at a donor level. In addition, it will allow us to use stricter thresholds for the analysis, similar to what has been used in single-cell eQTL or bulk sQTL studies to improve the results.

Larger single cell sQTL studies are likely to further demonstrate that regulation of expression and splicing are largely disconnected at the genetic level. We conjecture that this will hold true not just for the pancreatic islets, but for other conditions and tissues more generally. The increased number of variants associated with isoform choice will most likely facilitate the interpretation of the many GWAS variants where we lack a mechanistic understanding of how they influence the phenotype. Studies of disease conditions, such as T2D, will be particularly helpful for interpreting GWAS variants, and they are likely to further our understanding of the origins and role of cellular heterogeneity in health and disease (Benninger and Kravets, 2022). Taken together, our results provide a proof of concept that it is possible to identify cell type level sQTLs, and we envision that better powered studies will allow us to further uncover the genetic regulation of splicing.

## Data Availability

The original contributions presented in the study are included in the article/Supplementary Material, further inquiries can be directed to the corresponding author.
