# Supplementary material for: Single-cell splicing QTL analysis in pancreatic islets
Source: Front Bioinform. 2025 Sep 10;5:1657895. doi: 10.3389/fbinf.2025.1657895 (PMC12457394; doi:10.3389/fbinf.2025.1657895)
Supplement: Supplementary file 1 [file Supplementaryfile1.docx]

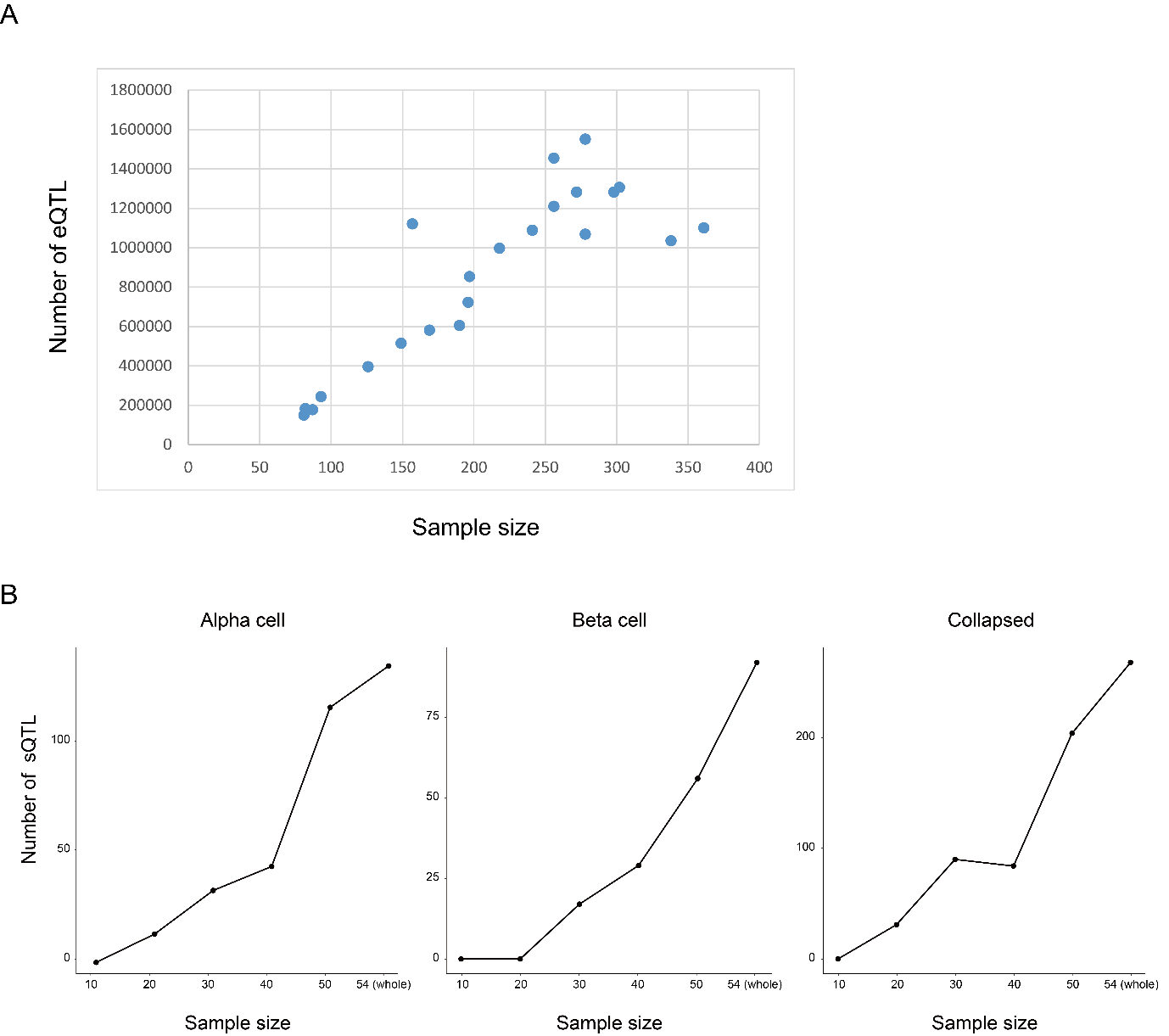


Supplementary Figure1.

(A) The scatter plot between the sample size and the number of cis-eQTL in GTEX data.

(B) The scatter plot between the sample size by down-sampling and the number of sQTL.


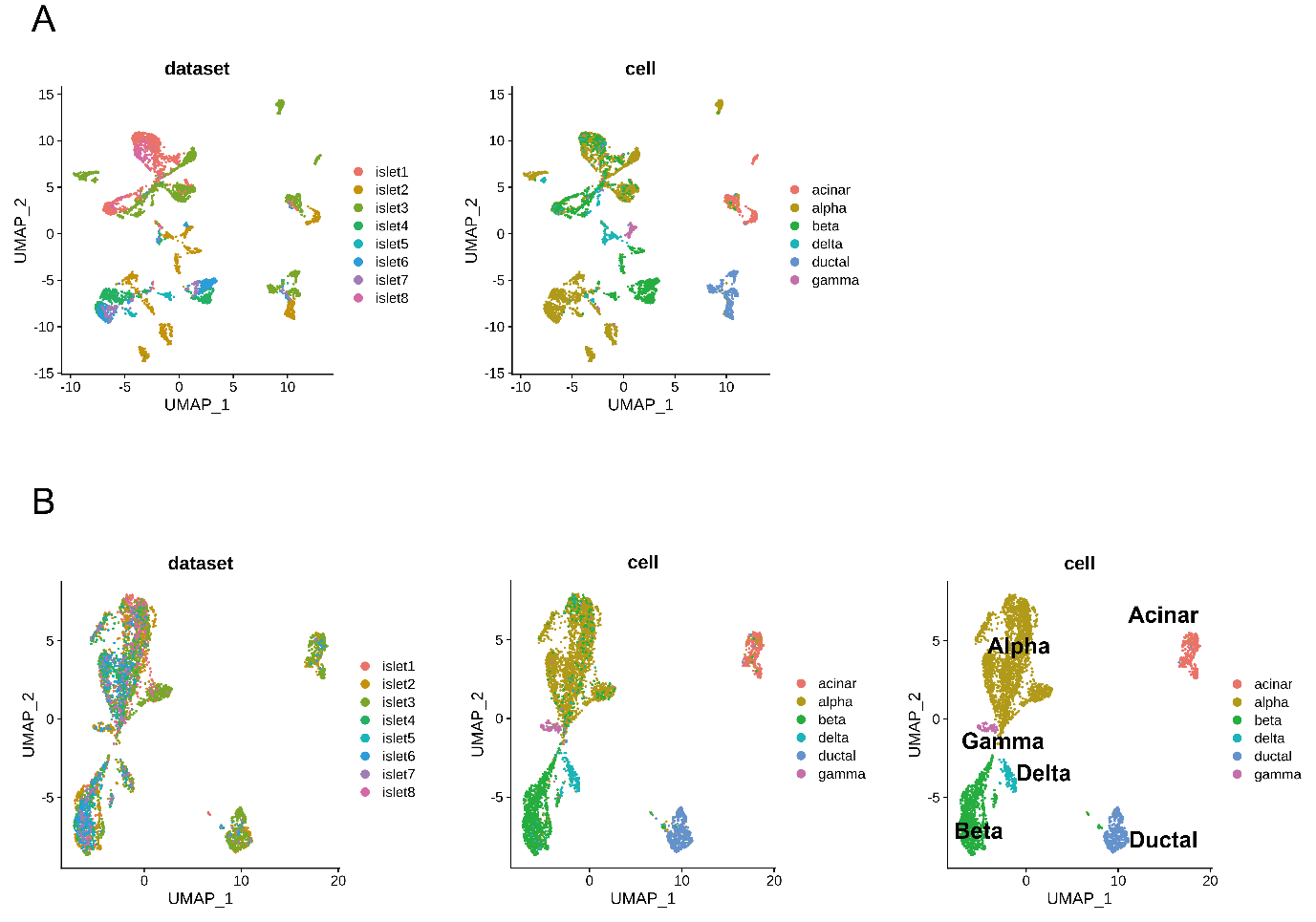


Supplementary Figure2. UMAP plot of islet atlas

(A) UMAP plot of islet atlas before batch correction in each dataset (left) and in each cell type (right)

(B) UMAP plot of islet atlas after batch correction (harmony). (left: each batch, middle: cell type before filtering, right: cell type after filtering)


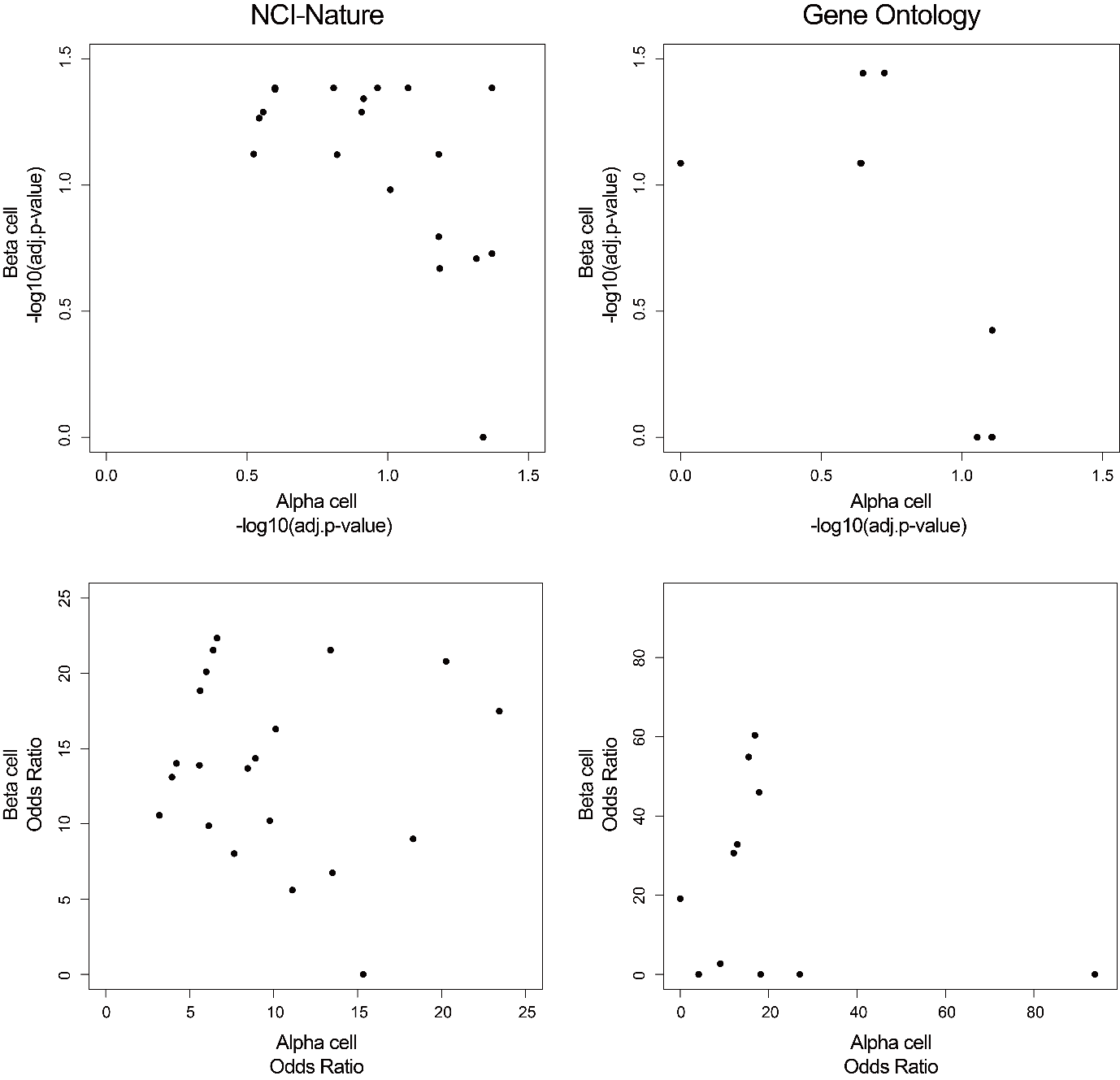


Supplementary Figure3. Scatter plot of pathway analysis from alpha and beta cells

Scatter plots show -log10 (adjusted p-value) (upper panel) and “Odds ratio” (lower panel) of the pathway enrichment analysis by EnrichR from alpha and beta cells. Only the pathways with adjusted p-value < 0.1 from either alpha or beta cells are shown.
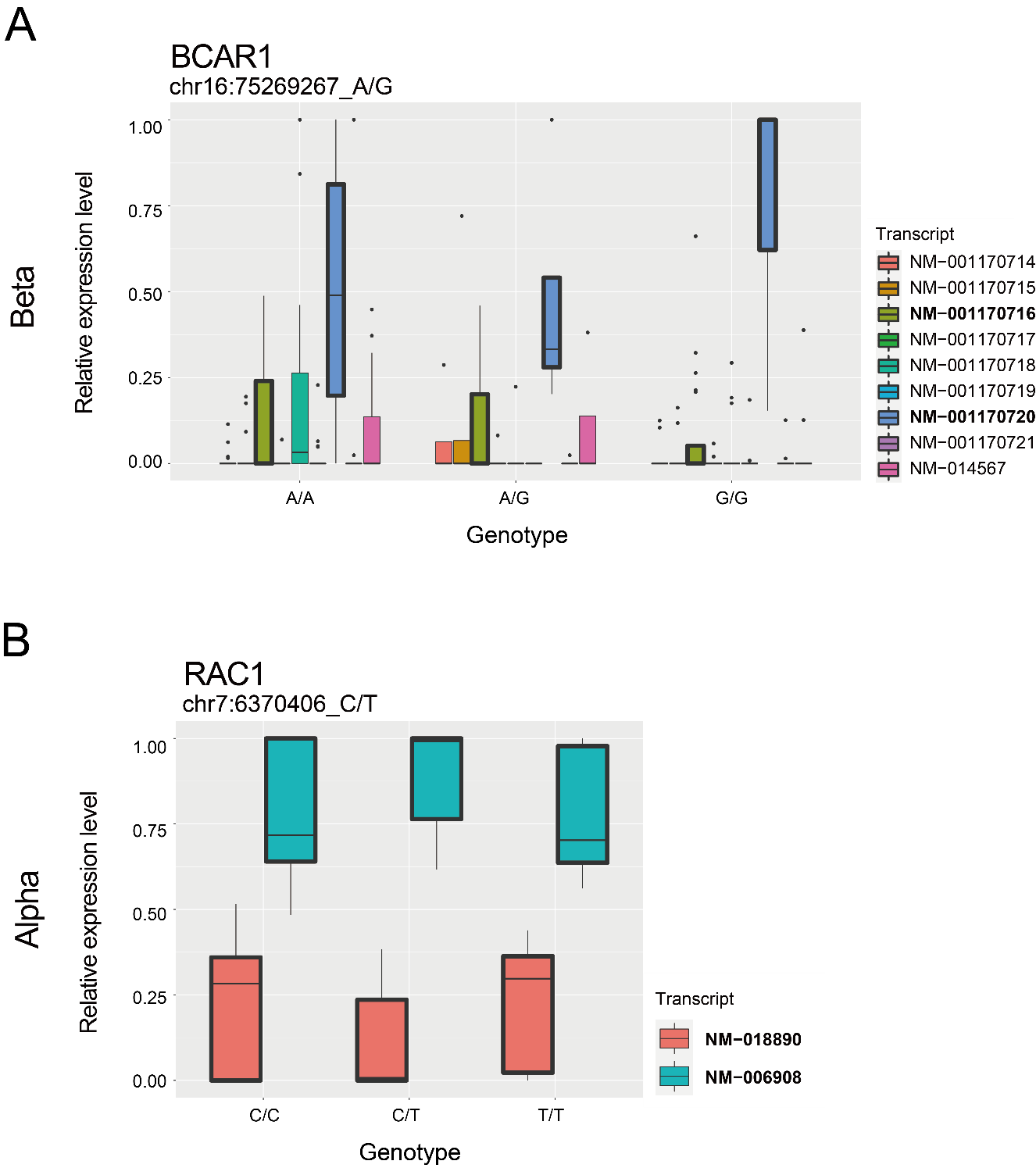


Supplementary Figure4. Distribution of relative transcripts in BCAR1 and RAC1 gene

(A) Relative abundance of each transcript (log2(exp+1)) at the BCAR1 locus for different genotypes in beta cells.

(B) Relative abundance of each transcript (log2(exp+1)) at the RAC1 locus for different genotypes in alpha cells.

Supplementary Figure5. The plot shows the number of sQTLs across the different thresholds of cell count for each donor in the alpha (left panel) and beta cells (right panel). If a donor has more cells than the threshold, we down-sampled the cells into a given threshold (Maximum cell count for alpha cell: 225, beta cell: 80).

**Supplementary Tables**

Supplementary Tables can be accessed via https://doi.org/10.5281/zenodo.14013685

Supplementary Table1

- metadata

Supplementary Table2

- genotype

Supplementary Table3

- cell_count

Supplementary Table4

- pseudobulk_transcript_matrix

Supplementary Table5

- sqtlseeker2_result

Supplementary Table6

- healthy_sqtl_fdr_0.1

Supplementary Table7

- SNP location information

Supplementary Table8

- eQTL

Supplementary Table9

- RBP analysis

Supplementary Table10

- splicetype

Supplementary Table11

- pathway
